# IgG4-related lymphadenopathy: A misdiagnosed case report

**DOI:** 10.1097/MD.0000000000047482

**Published:** 2026-01-30

**Authors:** Long Wang, Zhongjiao Chen, Deyu Guo

**Affiliations:** a Department of Pathology, Guiqian International Hospital, Guiyang, Guizhou, China.

**Keywords:** case report, IgG4-related disease, IgG4-related lymphadenopathy, misdiagnosis

## Abstract

**Rationale:**

IgG4-related lymphadenopathy (IgG4-RLP), a challenging diagnostic entity within IgG4-related disease (IgG4-RD), often faces delays in treatment due to frequent misdiagnosis.

**Patient concerns:**

A 53-year-old male presented with a 4-year history of a right upper arm mass and axillary lymphadenopathy. Initial investigations suggested alternative diagnoses, resulting in a prior misdiagnosis of reactive lymph node hyperplasia. The patient presented with enlarged axillary lymph nodes. Imaging showed cortical thickening, loss of cortico-medullary differentiation, and homogeneous enhancement on CT. Routine lab results were unremarkable, but ultrasound revealed multiple enlarged lymph nodes.

**Diagnoses:**

A multidisciplinary approach, including core needle biopsy and retrospective analysis, confirmed systemic IgG4-RLP. The diagnosis included a unique “checkerboard-like” fibrosis pattern in a prior specimen, and elevated IgG4 levels.

**Interventions:**

The patient was treated with methylprednisolone.

**Outcomes:**

The patient experienced significant symptom relief, correlating with a decrease in serum IgG4 levels, after initiating steroid treatment, at a 2-week follow-up.

**Lessons:**

Diagnosing IgG4-RLP is challenging, especially when encountering atypical histological patterns such as “checkerboard-like” fibrosis. Therefore, unexplained lymphadenopathy characterized by increased plasma cells should prompt high suspicion for IgG4-RLP. Prompt recognition through integrated clinical and laboratory findings is crucial to prevent misdiagnosis and ensure timely intervention.

## 1. Introduction

IgG4-related lymphadenopathy (IgG4-RLP) is a subtype of IgG4-related disease (IgG4-RD), which can manifest as localized or systemic lymphadenopathy, with or without involvement of extranodal tissues.^[1]^ When IgG4-RLP presents as the initial and only clinical manifestation, its pathological features may lack the typical hallmarks of IgG4-RD, such as IgG4 + plasma cell infiltration, fibrosis, and obliterative phlebitis, leading to confusion with conditions like lymphoma and infections. This can result in misdiagnosis and delayed treatment.^[2]^ We aim to enhance clinical awareness of this disease by reporting a case that was initially misdiagnosed but eventually confirmed as IgG4-RLP affecting systemic lymph nodes.

## 2. Case report

A 53-year-old Chinese male patient was again admitted to our hospital on June 14, 2025, with a chief complaint of a right upper arm mass of over 4 years’ duration. The patient reported that the mass originated approximately 4 years prior as a small, bean-sized nodule on his right upper arm, and had progressively enlarged to its current size, approximately the size of a pigeon’s egg. He noted associated aching pain which was aggravated by activities involving the arm, particularly lifting and extending objects. Of note, the patient had a history of a similar lesion in his left upper arm. In April 2020, he underwent excision of the lesion from our hospitalization, which was diagnosed pathologically as reactive lymph node hyperplasia. This prior lesion required no further intervention after comprehensive exclusion of significant findings regarding hereditary disease, infectious exposure, or animal contact.

On admission, physical examination revealed a mobile, nontender, and firm bean-sized nodule in the right axilla. Further palpation identified 2 subcutaneous masses on the medial aspect of the middle to lower right upper arm, measuring approximately 30 mm × 30 mm and 10 mm × 1 mm, respectively. Elbow extension was notably limited, confined to a range of 10° to 110°. Routine laboratory results, including the complete blood count, were unremarkable. Ultrasound showed multiple enlarged lymph nodes in both axillae and supraclavicular regions. The largest lymph node, in the axilla, measured 33 mm × 17 mm with a regular shape, clear boundaries, indistinct cortico-medullary differentiation, and cortical thickening. A CT scan of the right upper extremity further delineated multiple round or oval soft tissue masses within the subcutaneous tissue and axilla. The largest axillary mass (approximately 33 × 24 mm) showed uniform high signal intensity on T2-weighted imaging and isointense signal on T1-weighted imaging.

**Figure 1.**
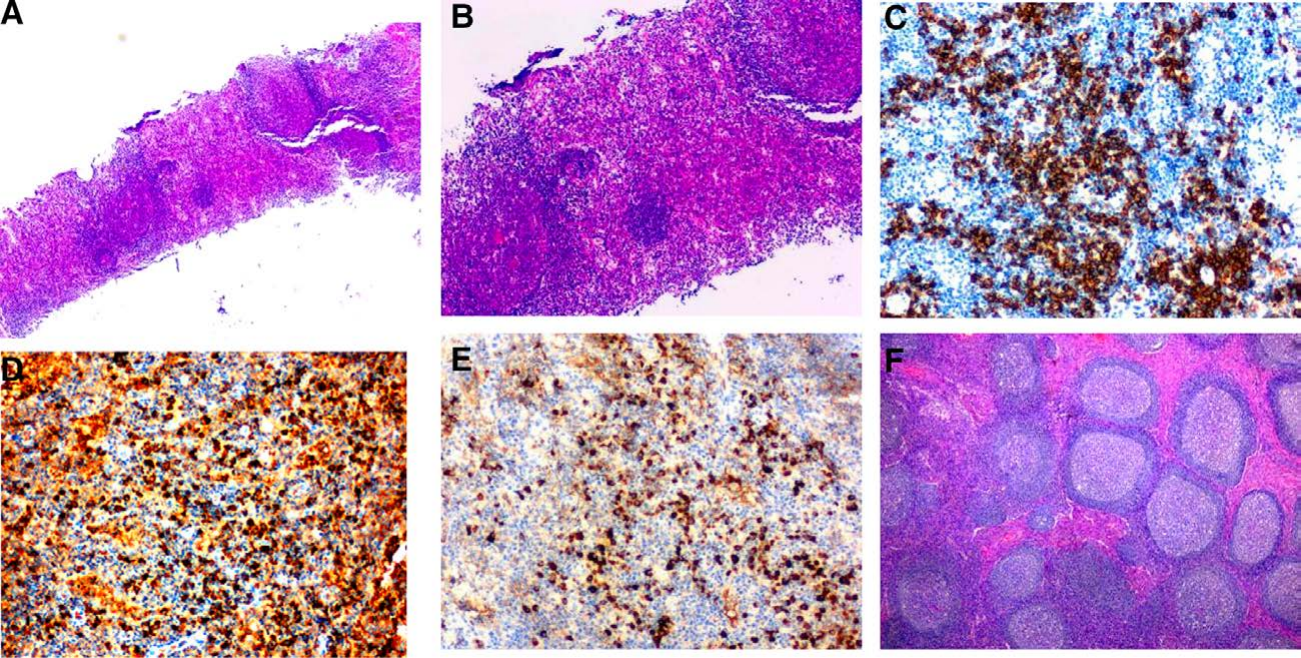
(A) Reactive follicular hyperplasia is evident, characterized by expanded interfollicular areas. (Hematoxylin and Eosin stain (H&E) 20 × magnification). (B) Abundant plasma cell infiltration along with scattered eosinophils in the interfollicular regions (H&E 40 × magnification). (C–E) Immunohistochemistry shows plasma cells expressing CD138 (C), IgG (D), and IgG4 (E). Specifically, more than 70% of plasma cells expressed IgG4, and over 80 IgG4-positive plasma cells were observed per high-power field. (100 × magnification) (F) Lymph node specimen from 5 years priorwith an IgG4/IgG ratioor revealing prominent interfollicular fibrosis with a characteristic “ checkerboard-like” fibrosis.(H&E 20 × magnification).

To determine the specific nature of the lesion, a core needle biopsy was conducted on a right axillary lymph node. The gross specimen comprised 2 gray-white tissue cores, each measuring approximately 15 mm × 2 mm. Microscopic examination of H&E-stained sections (refer to Fig. 1A and B) revealed preserved lymphoid follicle structures. However, significant expansion of the interfollicular areas was observed, characterized by a prominent density of plasma cells, eosinophils, and a paucity of neutrophils. This histological pattern strongly suggested a reactive process within the lymph node. To further delineate the cellular composition, immunohistochemical (IHC) staining was performed. Staining for germinal center markers, including CD20, CD10, and BCL6, showed characteristic positivity within the germinal centers, indicating their reactive nature. The absence of BCL2 positivity in the germinal center further supported a reactive, rather than neoplastic process. Furthermore, extensive infiltration by IgG- and IgG4-positive plasma cells was observed in the interfollicular regions (Fig. 1C–E), with an IgG4/IgG ratio exceeding 70% and over 80 IgG4-positive plasma cells per high-power field. Based on these pathological characteristics, a diagnosis of IgG4-RLP was considered, and serological testing was thus recommended. The results supported this diagnosis, as confirmed by subsequent serological analysis which revealed a significantly elevated IgG4 level of 456 mg/dL.

Is the diagnosis of suspected IgG4 RLP established? we retrospectively reviewed the patient’s archived left arm surgical specimen excised in September 2020 and performed immunohistochemical staining. H&E staining showed disrupted lymph node architecture, reactive changes in lymphoid follicles, and widespread interfollicular fibrosis with a characteristic “checkerboard-like” fibrosis (Fig. 1F). Numerous IgG4-positive plasma cells were identified within the fibrotic tissue (IgG4/IgG ratio of 80%, >120 IgG4-positive plasma cells per high-power field); no significant eosinophils were observed. In summary, considering the clinical presentation, radiological findings, significantly elevated serum IgG4 level, and histopathology, a diagnosis of IgG4-RLP was confidently made.

Methylprednisolone treatment was initiated, as it is a well-established first-line therapy for this condition. At a 2-week follow-up, the patient reported significant symptom relief, correlating with a decrease in serum IgG4 levels to 256 mg/dL. Consequently, the patient was advised to continue the prescribed medication and schedule follow-up appointments.

## 3. Discussion

The isolated presentation of IgG4-RLP as systemic lymphadenopathy, without concurrent extranodal involvement, is uncommon. In certain instances, lymph node diameters may reach up to 50 mm, and a minority of cases exhibit rapid disease progression, thereby increasing diagnostic complexity.^[3]^ While imaging modalities can offer suggestive clues for IgG4-RLP – for example, ultrasound may show cortical thickening and loss of cortico-medullary differentiation, and CT/MRI may reveal well-defined, uniformly enhancing lymph nodes lacking necrosis – these findings are nonspecific. Consequently, they are insufficient for reliable differentiation from other lymph node pathologies such as lymphoma, reactive lymph node hyperplasia, or Castleman disease.^[4]^ In this specific patient, imaging results, including axillary lymph node enlargement with compromised cortico-medullary differentiation, cortical thickening, and homogeneous enhancement on CT, were indeed consistent with the characteristic imaging features of IgG4-RLP. However, a conclusive diagnosis invariably necessitated pathological evidence.

For the diagnosis of IgG4-RLP, histopathological examination remains the definitive gold standard. While some scholarly works categorize IgG4-RLP into 5 histological subtypes, the full diagnostic import of these classifications has yet to be comprehensively understood.^[5]^ In this particular case, analysis of the axillary lymph node core biopsy demonstrated reactive lymphoid follicular hyperplasia, broadening of the interfollicular areas, and an infiltrate rich in plasma cells and eosinophils, along with a sparse number of neutrophils. Immunohistochemistry confirmed an IgG4/IgG ratio of approximately 70% (>80 IgG4-positive plasma cells/HPF), aligning with the histological features of the interfollicular widening subtype of IgG4-RLP, an indication of IgG4-RD.

Crucially, a histopathological review of the left arm lymph node, surgically removed 5 years earlier, revealed a complete loss of normal lymph node architecture and reactive changes within lymphoid follicles. However, this old specimen also exhibited extensive interfollicular fibrosis, creating a unique “checkerboard-like” fibrosis, heavily infiltrated by IgG4-positive plasma cells (IgG4/IgG ratio of 80%, >120 IgG4-positive plasma cells/HPF). Compared to the inflammatory pseudotumor-like changes frequently observed in IgG4-RD, the “checkerboard-like” fibrosis presents a distinctive pattern. This morphological characteristic might offer valuable differentiative information for this condition. Further research is needed to confirm its diagnostic value and specificity. The distinct presence of these 2 pathological subtypes in the same patient at different time points strongly implies that IgG4-RLP’s pathological subtypes may reflect varying stages of disease progression, with the fibrotic pattern potentially signifying a later or more chronic state. Consequently, early and accurate diagnosis becomes critically important to impede the advancement of the disease.

Diagnosing IgG4-RD via fine-needle aspiration (FNA) biopsy presents inherent challenges due to the lack of universally established definitive diagnostic criteria.^[6]^ However, the performance of FNA biopsy is crucial as it aims to minimize the need for surgical lymph node resection, thereby preserving regional function. In this particular patient, while the FNA indicated characteristic interfollicular widening and elevated IgG4-positive plasma cells (IgG4/IgG ratio), its morphological findings also overlapped with those of reactive hyperplasia. Per consensus guidelines,^[7,8]^ the concurrent fulfillment of 2 criteria – an absolute number of IgG4 + plasma cells (>50 or > 100 cells per high-power field) and a relative proportion (>40% of all IgG + cells) – should be interpreted as “suspected histological features” of IgG4-RD. In such ambiguous cases, a comprehensive assessment, integrating clinical presentation and serological tests, becomes paramount to avoid both over- and under-treatment. Furthermore, pathologists must remain vigilant for obliterative phlebitis and eosinophilia. These features are critical diagnostic clues; however, their often subtle nature often leads to their oversight, posing a significant risk of misdiagnosis. The sparse presence of neutrophils in this specific case might be related to local irritation, and its diagnostic significance is equivocal, potentially stemming from ongoing local stimulation.

Diagnosing IgG4-RD in a real-world clinical setting necessitates careful clinicopathological integration. Although elevated serum IgG4 levels are frequently observed in IgG4-RD, they lack adequate sensitivity or specificity. Thus, the diagnosis requires careful differential diagnosis, as serum IgG4 levels can be normal in affected patients or elevated due to alternative causes like chronic inflammation, infection, or neoplasms. If exclusion criteria are present (clinical, serological, imaging, or pathological), such as fever or histopathological evidence of granulomatous inflammation or necrosis, other diagnoses should be actively considered.^[7,8]^ For this patient, the elevated serum IgG4 level (456 mg/dL), when considered alongside the extensive infiltration of IgG4-positive plasma cells revealed in the lymph node pathology, provides strong diagnostic support for IgG4-RD. A brisk decline in serum IgG4 levels in response to treatment generally indicates a positive outcome. However, it is important to note that serum levels might not completely revert to normal even after achieving clinical remission, likely due to continued IgG4 production by long-lived plasma cells.

Differentiating IgG4-RLP from other conditions is critical, requiring careful exclusion of lymphoma, Castleman disease, and a variety of less common entities such as reactive lymph node hyperplasia, Rosai-Dorfman disease, and intranodal myofibroblastoma.^[9,10]^ Lymphoma (including Hodgkin and non-Hodgkin subtypes) is a primary concern due to its potential for rapid progression and associated B symptoms; pathological biopsy and molecular analysis are essential for definitive diagnosis. Castleman disease, particularly its multicentric form, may also present with systemic lymphadenopathy; however, its distinctive vascular proliferation and germinal center involution/hyalinization distinguish it from IgG4-RD; significant elevations in serum IgG4 are characteristically absent. Turning to the more common differential, reactive lymph node hyperplasia is more frequently encountered in the context of infection or chronic inflammation; careful assessment of serological and clinical data facilitates its differentiation from IgG4-RLP. Intranodal myofibroblastoma, a key differential, features spindle myofibroblastic proliferation with positive Vimentin, Desmin, SMA, and CD34; its typical presentation is that of a localized lesion without systemic features. In stark contrast, IgG4-RLP is characterized by extensive infiltration by IgG4-positive plasma cells, an elevated IgG4/IgG ratio, and frequent systemic involvement accompanied by elevated serum IgG4.

Although IgG4-RD typically follows an indolent course, early and accurate diagnosis remains crucial, as it can effectively delay tissue fibrosis and prevent organ dysfunction.^[11]^ Some patients may have asymptomatic but critical or life-threatening lesions, such as retroperitoneal fibrosis, periaortitis, and coronary arteritis. Therefore, early diagnosis is of paramount importance. Currently, glucocorticoids (steroids) remain a primary treatment for IgG4-RD. In this case, the patient’s symptoms significantly improved and serum IgG4 levels decreased after methylprednisolone treatment, suggesting effective therapy. The initial treatment dose and duration with glucocorticoids must be determined based on disease severity and organ involvement. During steroid treatment, it is crucial to regularly monitor blood glucose levels and consult with a diabetes specialist as needed. However, the high recurrence rate of IgG4-RD poses a significant clinical challenge that necessitates sustained management strategies to control disease reactivation. Therefore, immunosuppressants such as azathioprine and methotrexate are often used to maintain remission after steroid induction, helping to lower maintenance steroid doses and mitigate steroid-related adverse reactions. Additionally, monoclonal antibody therapies targeting B cells, such as rituximab, have shown promise in refractory or relapsing IgG4-RD patients, providing potential alternatives to long-term steroid use.^[9]^

## 4. Conclusion

IgG4-RD is highly treatable. However, its diagnostic complexity and the risk of misdiagnosis, particularly with IgG4-RLP, demand vigilance from clinicians and pathologists. This case highlights the critical need to consider IgG4-RD in the differential diagnosis of unexplained upper arm masses with lymphadenopathy. Achieving early, accurate diagnosis hinges on a multidisciplinary approach encompassing clinical assessment, imaging, serology, and histopathology, which is essential for personalized treatment and improved patient outcomes. Future research should focus on elucidating IgG4-RLP’s morphological complexity and pathological evolution across disease stages. These advancements, coupled with heightened clinical and pathological knowledge, will ultimately enhance early diagnosis and patient outcomes by mitigating steroid toxicities and preventing relapse.

## Author contributions

**Conceptualization:** Long Wang.

**Data curation:** Zhongjiao Chen.

**Investigation:** Long Wang.

**Writing – original draft:** Long Wang, Zhongjiao Chen.

**Writing – review & editing:** Long Wang, Deyu Guo.
